# A review of antimicrobial resistance in East Africa

**DOI:** 10.4102/ajlm.v5i1.432

**Published:** 2016-09-15

**Authors:** Lucas Ampaire, Abraham Muhindo, Patrick Orikiriza, Juliet Mwanga-Amumpaire, Lisa Bebell, Yap Boum

**Affiliations:** 1 Department of Medical Laboratory Sciences, Mbarara University of Science and Technology, Mbarara, Uganda; 2 Epicentre Mbarara Research Centre, Mbarara, Uganda; 3 Faculty of Medicine, Mbarara University of Science and Technology, Mbarara, Uganda; 4 Massachusetts General Hospital, Boston, Massachusetts, United States

## Abstract

**Background and objectives:**

Knowledge of local and regional antimicrobial resistance (AMR) is important for clinical decision making. However, surveillance capacity for AMR is lacking throughout East Africa, and current AMR data are sparse. We sought to address this gap by summarising all available high-quality data on AMR in the East Africa region.

**Method:**

We searched the PubMed database and African Journals Online archives in April and May 2015 using the search term ‘antimicrobial resistance AND sub-Saharan Africa’ to find articles published from 2005 onwards. Only full-text articles in English were included.

**Results:**

We included 12 published articles in our analysis. Most articles were on bloodstream infections, hospital-based and cross-sectional in design; a majority described either community- or hospital-acquired infections. High levels of AMR to commonly-used antibiotics were reported, including 50% – 100% resistance to ampicillin and cotrimoxazole infections, emerging resistance to gentamicin (20% – 47%) and relatively high levels of resistance to ceftriaxone (46% – 69%) among Gram-negative infections. Much of the resistance was reported to be in *Klebsiella* species and *Escherichia coli.* Among Gram-positive infections, extensive resistance was reported to ampicillin (100%), gentamicin and ceftriaxone (50% – 100%), with methicillin-resistant *Staphylococcus aureus* prevalence ranging from 2.6% – 4.0%.

**Conclusion:**

Overall, bacterial resistance was reported among commonly-used antibiotics (ampicillin, gentamicin and ceftriaxone), raising concern that these antibiotics may no longer be useful for treating moderate or severe bacterial infections in East Africa. Thus, empirical treatment of bacterial infections needs to be reconsidered and guided by local assessment of AMR. Improvements in the limited amount of quality data and lack of harmonisation in assessing the burden of AMR are also needed.

## Introduction

Without urgent, coordinated action by many stakeholders, the world is headed for a post-antibiotic era, in which common infections and minor injuries which have been treatable for decades can once again kill. (Dr Keiji Fukuda, WHO Assistant Director-General for Health Security)

The benefits of appropriate antibiotic use to treat bacterial infections are well established, although all antibiotic use carries a risk of inducing antimicrobial resistance (AMR). Throughout East Africa there is a heavy burden of community-acquired infectious disease.^1^ Unfortunately, the surveillance capacity for AMR is minimal in most East African countries, and current data on AMR patterns of common pathogenic bacteria are sparse.^2^ In addition, World Health Organization (WHO) surveillance reports indicate that there is inadequate coordination and harmonisation, compromising the ability to assess and monitor the situation.^3^ As a result, in these resource-constrained settings, the choice of antibiotic is often not based on known bacterial susceptibilities. Limited capacity for microbiology testing in East Africa coupled with a high burden of life-threatening bacterial infections reinforces a pattern of antibiotic prescription that is largely empirical, where AMR is detected only by therapeutic failure. Compounding the problem is the small repertoire of antimicrobials available in these settings, which are often of poor quality, when not counterfeit. In addition, in low-resource settings, antibiotics are often sold over the counter with minimal product regulation, oversight or quality control.^4^ The above, coupled with poor hygiene and infection control practices, may also spread community and/or hospital-acquired drug-resistant pathogens, further exacerbating the problem.^5^

The WHO global report^3^ on AMR indicates that resistance of common bacteria has reached alarming levels in many parts of the world. Furthermore, the report shows high proportions of resistance to third-generation cephalosporins and carbapenems: up to 54% among *Escherichia coli* and *Klebsiella pneumoniae*. Unfortunately, in East Africa, few good studies exist documenting the extent of AMR. The Global Antibiotic Resistance Partnership conducted by the Uganda National Academy of Sciences recently revealed worsening trends of resistance and diminishing effectiveness of antibiotics in Uganda.^6^ Some affordable drugs, such as penicillin G and cotrimoxazole, have been reported to have resistance at or near 100%.^6^

Although such reports are concerning, the burden of AMR in the East Africa region is not well published. Additionally, knowledge of the situation in many parts of the world further complicates the problem.^3^ Better knowledge of the burden and proportion of infections caused by drug-resistant bacteria in low-resource settings would raise awareness of the need to prevent the rise and spread of drug resistance. Understanding current levels of AMR throughout East Africa could improve clinical practice by guiding empirical antibiotic choice. Toward this end, we reviewed the available evidence on the burden of AMR among bacterial pathogens in East Africa in order to inform current clinical practice and future research interventions to address antibiotic resistance.

## Methods

### Literature review

We searched the PubMed database and African Journals Online archives in April and May 2015. We used the term ‘antimicrobial resistance AND sub-Saharan Africa’ to find articles published from 2005 onwards. Only articles in English were included.

### Study selection criteria

Full-text articles were included if they reported the proportion of antibiotic resistance among clinical isolates of pathogenic bacteria collected from inpatients and outpatients in any of the following East African countries: Uganda, Kenya, Tanzania, Rwanda, Ethiopia and Democratic Republic of Congo. Eligible studies were required to describe the patient population studied, organisms isolated, specific laboratory methods used for the determination of pathogen antimicrobial sensitivity patterns, and an interpretation of the specific minimum inhibitory concentration breakpoints or the diameter of the zone of inhibition of the antibiotics tested as described by the Clinical and Laboratory Standards Institute.^7,8^ Both adult and paediatric patient populations were included, but case reports were excluded from the review as has been done previously.^9^ For overlapping studies reporting on the same clinical isolates, only the study with the largest sample size was included. In an effort to incorporate contemporary, relevant AMR data, only studies published from 2005 onwards were included in the review.

### Data extraction

The extracted data included bacterial species isolated, the number of isolates tested for AMR, specific antibiotics tested for resistance, and percentage of organisms resistant to each antibiotic. Extracted data were grouped on the basis of whether they caused bloodstream infections or other infections.

## Results

**FIGURE 1:**
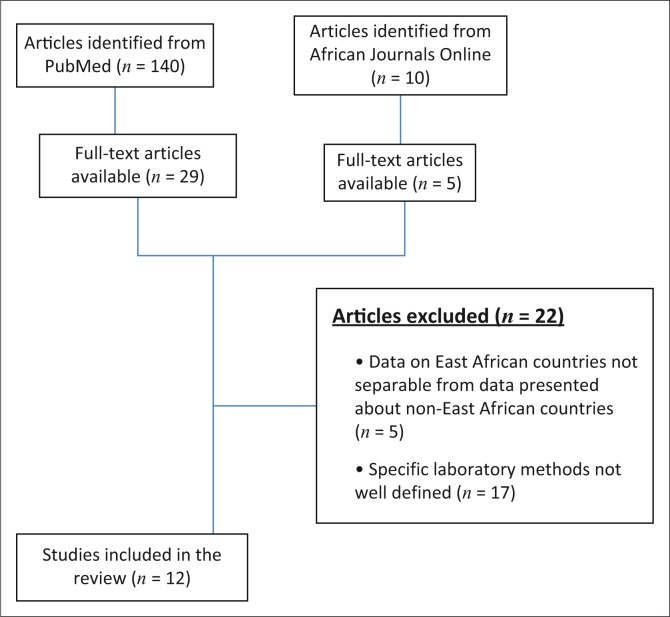
Selection of publications for inclusion in this review.

We initially identified 150 articles: 140 from PubMed and 10 from African Journals Online. Full-text articles were available for 34 papers identified by the search. Of the remaining 116 manuscripts, only abstracts were freely available for 105 articles; 11 presented information on anti-tuberculosis drug resistance and lacked information on non-mycobacterial infections, which rendered them ineligible for inclusion in this review. Of the 34 full-text articles available, 22 were excluded, because the data presented were from non-East African countries that were inseparable from data presented about East African countries (*n* = 5) or their laboratory methods were not well defined (*n* = 17) (Figure 1). The remaining 12 articles were included in this review, six describing AMR patterns in Uganda,^10,11,12,13,14,15^ five in Ethiopia^16,17,18,19,20^ and one in Tanzania^21^ (Table 1). Neither studies from Rwanda nor studies from the Democratic Republic of the Congo met inclusion criteria for this review. All studies were hospital-based and cross-sectional in design, and the majority described both community- and hospital-acquired infections. Four studies presented data on bloodstream infection, seven focused on other infections excluding bloodstream infections and one reported clinical specimens from multiple anatomical sites.

**TABLE 1:** Summary of East Africa-based studies included in the analysis.

Country	Year	Bacterial species (*N*)		Patient population	No. of patients	Reference No.
		Gram-positive	Gram-negative			
**Patients with bloodstream infections**						
Uganda	2006	31	13	Severely malnourished, HIV-positive children	450	13
Uganda	2006	76	20	Neonates with confirmed septicemia	293	10
Ethiopia	2013	17	17	Children and adults with suspected septicemia	390	17
Tanzania	2005	66	103	Children with fatal septicemia	1798	21
**Patients with other, non-bloodstream infections**						
Ethiopia	2012	12	22	Pregnant women with UTIs	367	16
Ethiopia	2012	-	22	Post-operative nosocomial infections	294	19
Ethiopia	2014	-	21	UTI in obstetric fistula patients	53	18
Ethiopia	2014	-	35	Stool isolates in children < 5 years with diarrhoea	158	20
Uganda	2011	66	-	MRSA from surgical patients	122	11
Uganda	2013	95	209	Surgical hospitalised patients	314	14
Uganda	2009	-	123	*Neisseria gonorrhoeae* isolates from sex workers	123	15
Uganda	2013	300	-	Clinical isolates of *Staphylococcus aureus* from various clinical specimens	300	12

MRSA, methicillin-resistant *Staphylococcus aureus*; UTI, urinary tract infection.

### Antimicrobial resistance patterns

#### Bloodstream infections

According to the disk diffusion methods used in studies included in this review, pathogens exhibited relatively high levels of resistance to antibiotics commonly used in East Africa. For Gram-negative organisms (Table 2), 50% – 100% resistance was reported to ampicillin and cotrimoxazole, two of the most frequently-prescribed antibiotics in this region.^16^ High levels of resistance to ampicillin among children with bloodstream infections (75% – 100%)^7,10,16^ were also reported, as were lower, but significant, levels of resistance to gentamicin (20% – 47%).^7,10,16^ Among Gram-positive organisms isolated from bloodstream infections, overall relatively low levels of resistance were reported to gentamycin, ampicillin and chloramphenicol (4% – 12%).^10,11^ However, studies reporting specifically on hospital-acquired strains noted high-level resistance to ampicillin, gentamycin, chloramphenicol and trimethoprim-sulfamethoxazole (29%).^11,19^

**TABLE 2:** Antibiotic resistance patterns among patients with bloodstream infections.

Bacteria	No. of species	Percentage resistant by Kirby Bauer disk diffusion method						Country	Reference
		AMP	SXT	CHLO	PEN	CRO	GENT		
**Gram-positive**									
*Staphylococcus aureus*	20	35	77	37	-	-	12	Uganda	11
	69	88	-	73	-	-	4	Uganda	10
	17	47	58	23	-	-	29	Ethiopia	17
	30	100	-	-	-	-	-	Tanzania	21
*Enterococcus faecalis*	9†	-	-	62	83	-	44	Tanzania	21
	6‡	-	-	67	25	-	33	Tanzania	21
*Enterococcus faecium*	12†	75	63	-	90	-	23	Tanzania	21
	9‡	89	100	-	100	-	77	Tanzania	21
*Streptococcus pneumoniae*	11	27	100	27	-	-	-	Uganda	13
*Streptococcus agalactiae*	7	14	-	14	-	-	57	Uganda	10
**Gram-negative**									
*Escherichia coli*	6	83	100	50	-	-	-	Uganda	13
	17	-	-	-	-	-	29	Uganda	10
	5	100	40	20	40	-	-	Ethiopia	17
	24†	96	-	-	-	22	29	Tanzania	21
	13‡	85	-	-	-	4	46	Tanzania	21
*Klebsiella* spp.	8	75	50	62	-	62	37	Ethiopia	17
	19†	100	-	-	-	26	47	Tanzania	21
	34‡	100	-	-	-	15	47	Tanzania	21
*Pseudomonas aeruginosa*	4	100	75	25	-	25	-	Ethiopia	17
	7†	100	-	-	-	80	24	Tanzania	21
	6‡	100	-	-	-	100	27	Tanzania	21
*Salmonella* spp.	5	80	80	-	-	-	20	Uganda	13
	3	-	-	-	-	-	33	Uganda	10
*Haemophilus* spp.	2	100	100	50	-	-	-	Uganda	13

AMP, Ampicillin; CRO, Cefuroxime; CHLO, Chloramphenicol; GENT, Gentamicin; PEN, Penicillin; SXT, Trimethoprim-sulfamethoxazole.

† Hospital-acquired

‡ Community acquired.

### Non-bloodstream infections

#### Surgical site infections

Among organisms isolated from surgical site infections in hospitalised patients, *Staphylococcus aureus* and coagulase-negative *Staphylococcus* were the most common Gram-positive organisms, whereas *Klebsiella* spp.*, Proteus* spp. and *E. coli* were the most common Gram-negative organisms^10,16,22^ (Table 3). Multiple studies reported 100% resistance to ampicillin for surgical site infections among hospitalised adults.^16,22^ There was also notable resistance of Gram-positive organisms to gentamicin and ceftriaxone in post-operative nosocomial isolates (50% – 100%), with methicillin-resistant *Staphylococcus aureus* (MRSA) prevalence ranging from 2.6% – 4.0%.^16^

**TABLE 3:** Antibiotic resistance patterns among patients with other, non-bloodstream infections.

Bacteria	No. of species	Percentage resistant by Kirby Bauer disk diffusion method													Country	Reference
		AMP	SXT	TTC	CHLO	ERY	GENT	AMX	CEFT	CIPRO	DOXY	F	KAN	CL		
**Gram-positive**																
*Coagulase-negative Staphylococci*	6	50	66	50	16	-	16	33	-	50	-	16	33	-	Ethiopia	16
	9	100	11	55	55	55	44	-	44	44	33	-	-	-	Uganda	11
*Staphylococcus aureus*	3	66	33	66	33	-	-	-	-	33	-	33	-	-	Ethiopia	16
	66	25	50	54.7	65.6	92.2	100	-	-	98.4	-	-	-	-	Uganda	11
	64	100	89	42	15	46	18	-	-	-	-	-	-	-	Uganda	14
	300	-	62	-	-	47	-	-	-	-	-	-	-	36	Uganda	12
*Streptococcus agalactiae*	2	50	50	50	50	-	50	50	-	50	-	-	50	-	Uganda	14
*Enterococcus* spp.	23	30	-	74	30	65	21	-	-	60	-	-	-	-	Uganda	14
**Gram-negative**																
*Escherichia coli*	16	81	56	43	56	-	31	75	-	18	-	-	63	-	Ethiopia	16
	9	78	67	66	-	-	-	90	55	44	66	22	44	-	Ethiopia	19
	6	67	67	100	17	-	67	50	50	50	-	-	-	-	Ethiopia	18
	72	100	81	72	41	-	54	-	77	72	-	-	-	-	Uganda	14
*Klebsiella pneumoniae*	39	100	92	76	71	-	76	-	92	66	-	-	-	-	Uganda	14
	2	100	100	-	100	-	100	100	100	100	50	50	50	-	Ethiopia	19
*Pseudomonas aeruginosa*	2	100	50	50	100	-	50	50	-	-	-	-	50	-	Ethiopia	16
	5	100	60	20	80	-	40	100	100	40	80	100	60	-	Ethiopia	19
	12	-	100	100	-	-	16	-	16	-	-	-	-	-	Uganda	14
*Proteus mirabilis*	5	100	60	20	80	-	40	100	100	40	80	100	60	-	Ethiopia	19
	2	50	50	-	50	-	-	100	-	-	-	100	-	-	Ethiopia	16
*Citrobacter freundi*	13	69	54	62	77	-	62	69	46	54	-	-	-	-	Ethiopia	18
	2	100	100	-	100	-	-	100	-	100	-	-	-	-	Ethiopia	16
*Salmonella* serogroup A	1	-	-	-	-	-	-	-	100	-	-	-	-	-	Ethiopia	20
*Salmonella* serogroup B	3	-	-	-	-	-	-	-	100	-	-	-	-	-	Ethiopia	20
*Shigella* spp.	11	63	-	54	9	90	27	100	55	-	-	-	-	-	Ethiopia	20
*Campylobacter* spp.	20	30	20	15	-	55	70	80	-	-	-	-	-	-	Ethiopia	20
*Acinetobacter* spp.	52	-	98	65	-	-	88	-	-	77	-	-	-	-	Uganda	14
*Neisseria gonorrhoeae*	123	-	-	-	-	-	-	-	-	81	-	-	-	-	Uganda	15

AMP, Ampicillin; AMX, Amoxicillin; CEFT, Ceftriaxone; CHLO, Chloramphenicol; CIPRO, Ciprofloxacin; CL, Clindamycin; DOXY, Doxycycline; ERY, Erythromycin; F, Nitrofurantoin; GENT, Gentamicin; KAN, Kanamycin; SXT, Trimethoprim-sulfamethoxazole; TTC, Tetracycline.

#### Urinary tract infections

High-level resistance to ampicillin (50% – 100%) was seen in urinary tract infections, where *E. coli* was the most common pathogen.^16,18^ *Citrobacter freundii* was a major cause of urinary tract infections in one study focused on obstetric fistula patients, with resistance to ampicillin, gentamicin, and ceftriaxone ranging from 46% – 69%.^18^ Other causative organisms of urinary tract infections, such as *Klebsiella* spp.*, Enterobacter* spp. and *Proteus* spp., also showed significant resistance to ampicillin, gentamycin and ceftriaxone, some of the most commonly-used antibiotics in East Africa.^16,18^ There was also high-level resistance reported of *Neisseria gonorrhoeae* to ciprofloxacin (81%) among sex workers in Uganda.^21^

#### Gastrointestinal tract infections

*Salmonella* spp. in stool isolates from children under five years of age demonstrated complete resistance to ceftriaxone (100%). From the gastrointestinal tract, the commonly isolated bacteria were *Campylobacter* spp. and *Shigella* spp. *Campylobacter* spp. showed moderate amounts of resistance to ampicillin (30%) and high-level resistance to gentamicin (70%). Among *Shigella* spp., relatively high numbers were resistant to ampicillin (63%), although resistance to gentamycin was lower at 27% in one Ethiopian study.^20^

#### Multiple body sites

One study from Uganda on phenotypic clindamycin resistance found that 109 (36%) of *S. aureus* isolates were resistant to clindamycin, of which 9 (3%) were constitutively resistant and 100 (33.3%) were inducibly resistant. In this study, *S. aureus* also showed significant resistance to trimethoprim-sulfamethoxazole (62%) and oxacilin (36%), with a demonstrated prevalence of MRSA equal to 36%.^12^ Resistance to vancomycin, one of the last-line drugs for treating MRSA, was also found (7.3%).^12^

## Discussion

In this review, we summarise the findings of 12 studies that demonstrate significant resistance across the East Africa region to antibiotics important for everyday use. Overall, although AMR varies throughout the region, we found that most Gram-negative organisms have limited susceptibility to ampicillin, ceftriaxone and gentamicin, which are commonly-used first-line empirical antibiotics in this region and recommended by the WHO Integrated Management of Childhood Illness for treatment of severely-ill infants. There was also significant resistance to cotrimoxazole, penicillins, quinolones, cephalosporins and aminoglycosides among Gram-positive organisms.

Several published papers we reviewed reported single bacterial isolates resistant to multiple antibiotics. Antibiotic resistance to multiple drugs was most common among Gram-negative organisms isolated from hospital-acquired infections in post-operative patients and hospitalised adults.^16,23^ However, the susceptibility of Gram-negative organisms to ciprofloxacin was generally reported to be good; thus, this may be the drug of choice for empirical use against post-operative nosocomial infections with Gram-negative organisms. The finding of multi-drug resistance in this population suggests that efforts to promote appropriate antibiotic use and microbiological sampling of infected patients should be targeted to these groups in low-resource settings.

The evidence presented here indicates that AMR, especially to the widely-used antibiotics (ampicillin, tetracyclines and trimethoprim-sulfamethoxazole), is prevalent and common in East Africa and may be a growing problem, especially among hospitalised and post-operative patients. However, resistance is likely under-reported in this region as noted by the WHO Global Report on AMR in 2014,^3^ due to limited availability of diagnostic testing, microbiology support and limited comparability of laboratory standards. Many of the same factors leading to the inability to test clinical isolates for antimicrobial susceptibility contribute to antibiotic overuse and misuse when laboratory data are lacking and can contribute to exacerbation of AMR.

Even when information about AMR is available, it may not be properly communicated to those prescribing medications in East Africa, due to inadequate national laboratory strategic plans throughout the region. In addition, guidelines regarding appropriate selection of drugs are inadequate.^11,18^ Compounding these problems is a lack of rigorous infection control procedures, all of which could lead to the development and spread of antibiotic resistant bacteria. These factors combine to support the spread of existing AMR throughout East Africa.

### Preventing the development and spread of antimicrobial resistance

AMR is likely to become an even greater problem in East Africa and may be exacerbated by overuse of antibiotics, the lack of oversight of antibiotic prescription, and the paucity of relevant local data on AMR. To address these issues, existing antimicrobial stewardship programmes should be strengthened or, where they are not yet in place, they should be developed and implemented in all regional referral hospitals in response to these challenges. Based on our findings, an area of particular focus should be hospitalised patients. Existing but limited resources should be directed equally at discovering the causes and at appropriate treatment of infections in this population.

Additionally, there is a need to urgently scale-up training of both laboratory and pharmacy staff in antibiotic stewardship at health facilities where laboratory investigations are available. This is critical in communicating with clinicians, who are the cornerstone of proper management of patients with infections, especially with regard to the use of antibiotics. There is also need to regularly conduct antibiotic resistance surveys to establish evidence-based and locally-relevant antibiotic resistance information that would be helpful in creating guidelines to improve clinical practice.

The implementation of the 2009 WHO global strategy for containment of AMR through inter-continental-wide surveillance programmes as a health systems approach^12,23,24,25^ has met with a number of challenges. In low- and middle-income countries, implementing the strategy has proven difficult, because human and financial resources and microbiology expertise are insufficient. In addition, it is difficult to obtain appropriate sample sizes for an accurate representation of resistance patterns. Novel approaches to antimicrobial surveillance are therefore needed for low-resource settings, which include the development of surveillance programmes utilising smaller sample sizes to provide locally-relevant AMR patterns and to encourage appropriate empirical antimicrobial therapy.^26^ Moreover, the development of new point-of-care diagnostic tools able to detect AMR in a cost-effective way will improve patient management and limit the emergence of drug resistance.

Lastly, from among the total of 150 publications we identified, we considered only 12 due to the lack of standardisation and quality of the methodology and reporting. This highlights the scarcity of good quality data that could allow stakeholders to assess the real burden of AMR. Thus, better standardised research protocols are needed to evaluate the emergence of AMR in different settings to obtain comparable results and implement tailor-made interventions.

### Conclusion

Based on the findings in this review, resistance to commonly-used antibiotics is prevalent in East Africa. Multi-drug resistance has been noted as a rising threat in the region and threatens to further complicate the drug resistance burden, especially for non-bloodstream infections where a single isolate may be resistant to more than one antibiotic drug of choice in different or similar drug lines.

Data from interventional studies designed to reduce AMR are particularly lacking in the East African context, where infectious disease prevalence is high. The profound lack of data on hospital-acquired infections and prevalence of AMR in low-income East African countries calls for vigorous investigation and surveillance to better define the problem. There is a need for countries to promote acceptance of antimicrobial stewardship as a programmatic strategy, including pharmacy management, laboratory quality control, complete microbiology investigations and creation and dissemination of regional standard antibiograms.
